# Vaccination, Risks, and Freedom: The Seat Belt Analogy

**DOI:** 10.1093/phe/phz014

**Published:** 2019-10-22

**Authors:** Alberto Giubilini, Julian Savulescu

**Affiliations:** 1 University of Oxford; 2 University of Oxford and Murdoch Children's Research Institute, Melbourne

## Abstract

We argue that, from the point of view public health ethics, vaccination is significantly analogous to seat belt use in motor vehicles and that coercive vaccination policies are ethically justified for the same reasons why coercive seat belt laws are ethically justified. We start by taking seriously the small risk of vaccines’ side effects and the fact that such risks might need to be coercively imposed on individuals. If millions of individuals are vaccinated, even a very small risk of serious side effects implies that, statistically, at some point side effects will occur. Imposing such risks raises issues about individual freedom to decide what risks to take on oneself or on one’s children and about attribution of responsibility in case of adverse side effects. Seat belt requirements raise many of the same ethical issues as vaccination requirements, and seat belt laws initially encountered some opposition from the public that is very similar to some of the current opposition to vaccine mandates. The analogy suggests that the risks of vaccines do not constitute strong enough reasons against coercive vaccination policies and that the same reasons that justify compulsory seat belt use—a measure now widely accepted and endorsed—also justify coercive vaccination policies.

## Prologue: A Story of Resistance to Compulsory Seat Belt Laws

‘We are proposing making it mandatory to wear a seat belt in cars for all occupants’, says a Government Official.

‘That would be wrong! It's a liberty issue. We should be free to decide whether to wear a seat belt or not - it's our body and choice. It is not the State's business’ replies the Libertarian.

‘It is a very small inconvenience to prevent a great harm. And it is not only yourself who is affected. It is your children who are in your care: we want them to be buckled up because it is in their best interest. And then there are third parties who will receive worse health care because of the costs you impose through your choices’, replies the Official.

‘The use of healthcare resources should not be a constraint on my liberty: I have the right to do what I want with my body and refuse interventions on it. Besides, seat belts can be dangerous. They can cause severe injuries. It can be safer to be thrown clear of an accident, rather than caught in it, and the state cannot decide what risks to impose on me or on my children’, says the Libertarian.

‘Seat belts can cause injuries but it is vastly more likely that they will protect you. It is all about probabilities and the chances are on the side of wearing seat belts’, replies the Official.

‘But I should be free to decide what risks to take for myself and my children. And it's annoying to try to put on the seat belt, kids want to play in the car, or sleep, and the risks are very small’.

‘It is true that the risks are small but if they materialise, the harms are enormous. It is just not worth it to be a quadriplegic’.

‘It will be expensive and difficult to enforce, with invasions of privacy. It is the beginning of a police state’, concludes the Libertarian.

Initially, compulsory seat belt laws met with great resistance, exemplified by this hypothetical, but realistic, dialogue; the main conflict of values was between individual (and parental) autonomy on one side, and favourable cost-benefit analysis and public interest on the other. As we shall see below, only 1 in 5 people in the US consistently used seat belts before mandatory legislations were passed, and in some states almost half of the population was opposed to mandatory seat belt laws. Within a few years, wearing seat belts became widely accepted and indeed endorsed in most countries. It became not only a legal, but also a social norm precisely because it was made compulsory and people started buckling up.

All of the same arguments against mandatory seat belt laws are often given by those who think that the state should not coerce people into vaccinating children. And all of the same replies can be given, and actually even stronger replies, since that failure to vaccinate is more likely than failure to buckle up to harm others. If choice were removed, vaccination might become a social norm, just as wearing seat belts is in those countries where they are compulsory, in spite of the small risks involved by vaccination. In this paper, we are going to expound this analogy and use it to defeat some objections to coercive vaccination polices. The upshot of our discussion is that, unless other objections can be raised, certain vaccinations may and should be coerced by the state.

A couple of clarificatory remarks are in order before we start. First, by ‘coercive’ vaccination policies we mean here policies that either make it illegal not to vaccinate, for example by fining parents of non-vaccinated children (as is currently the case in Italy), or that make certain goods contingent upon vaccinating one’s children, for example by excluding children from public school without any possibility of nonmedical exemption (as is the case in some US States, namely California, West Virginia, and Mississippi). Following Mark Navin and Mark Largent (Navin and Largent, 2017), we define these cases as ‘compulsory’ and ‘mandatory’ vaccination, respectively (although it is worth noting that this terminological distinction is normally not adopted in other contexts, and most notably with regard to seat belt laws, where ‘mandatory’ indicates that seat belts are legal requirements). Second, when we talk of vaccination in this paper, we refer to at least some of the vaccines that are normally recommended, mandated, or imposed in Western countries because the infectious diseases they protect from represent serious enough threats to individual and public health in those countries, and not to all possible vaccines available (thus, for instance, we would include the measles, mumps, and rubella (MMR) vaccine, and the flu vaccine, which we discuss in introduction section, but not the yellow fever vaccine).

## Introduction

Like almost any medical drug, vaccination entails some very small risk of more or less serious injury. The risk is extremely small and normally it is vastly outweighed by the benefits of vaccines. According to the WHO, ‘so few deaths can plausibly be attributed to vaccines that it is hard to assess the risk statistically’, and ‘serious adverse events occur rarely (on the order of one per thousands to one per millions of doses)’ (WHO, 2018). None of these phrases, however, rules out that in extremely rare circumstances vaccines can cause side effects, and sometimes significant ones. For instance, the flu vaccine can, in some very exceptional circumstances, cause Guillain-Barré Syndrome, and vaccines carry the risk of allergic reactions. One of the reasons why some people in Western countries are opposed to coercive vaccination policies is precisely the concern for the risks of vaccines (Salmon *et al.*, 2005; Smith *et al.*, 2011; Harmsen *et al.*, 2013), including the often mistaken belief that the risks of vaccines are greater than the risks of the infectious diseases they would prevent (Wang *et al.*, 2014).

That medical drugs involve some risks is normally not seen as particularly ethically problematic. Aspirin can in very rare cases cause bleeding in the stomach resulting in vomiting blood and bleeding in the brain resulting in stroke symptoms. Paracetamol can in rare cases cause significant blood disorders. Still, people normally use aspirin and paracetamol, which in some countries, such as the UK, can be bought over the counter at supermarkets. However, coercive vaccination policies raise more ethical concerns about possible injuries, for two reasons. For one thing, normally, individuals can decide whether or not to take on themselves the risks of medical interventions; I can choose to keep my headache or, more controversially, my child’s headache without incurring the small risks entailed by aspirin or paracetamol. Coercive vaccination policies remove to a significant extent the freedom to decide whether or not to take certain risks. For another thing, even when medical interventions are imposed because necessary to protect the best interests of children, such risks are normally justified by the existence of some condition that requires medical treatment; and even when they are not accepted by parents (for example, because they are committed to natural life-style or have certain religious views against certain medical interventions), most would agree that forced medical treatments for serious health conditions of vulnerable individuals are justified—think, for instance, of the case of blood transfusions for children of Jehovah’s Witnesses families.

With coercive vaccination policies, however, neither consideration applies: the risk of vaccine injuries is simply imposed on individuals, either adults or children, and it is imposed without any underlying medical condition that justifies their use. The lack of any pathology to be treated reinforces some parents’ perception that the risks of vaccines are not only great and greater than they actually are, but also unnecessary, as 74 per cent of non-vaccinating parents surveyed in the USA stated (Hough-Telford *et al.*, 2016). As a result, restriction of freedom of choice is perceived as unjustified. We should specify that opposition to coercive vaccination policies is not necessarily based on risk perception or on a negative attitude towards vaccines. Some people might consistently think that vaccines are overall a good thing and even that people have a moral obligation to be vaccinated, but be opposed to state coercion in the name of individual freedom or of some other value such as bodily integrity. Here, however, we will focus specifically on the liberty to decide which risks to take on oneself or one’s children and to decide what represents a risk worth taking. Are the small risks of vaccines and the value of freedom of choice (including in cases of misperception of risks) strong enough reasons against coercive vaccination policies? As we shall see in the next section, this question is worth considering because, where there are effective coercive vaccination policies, it might happen that sooner or later some children will experience some vaccine injury, perhaps even serious ones. Statistically, if millions of children are vaccinated, this is a very realistic scenario.

Needless to say, but perhaps worth emphasizing, there is no evidence to support the idea that the risk assessment of those who oppose vaccines—and on such basis oppose coercive vaccination policies—is rational. For instance, the flu vaccine, as mentioned earlier, might—and we say ‘might’ because the causal link is contested—entail a very small risk of causing Guillain-Barré Syndrome (GBS), a serious and sometimes incurable autoimmune disorder that can result in paralysis and death; the risk is in the order of 1–2 cases per million flu vaccine doses. But interestingly enough, also the flu can cause GBS, and actually the risk—although very small in both cases—is higher after catching the flu than after being vaccinated (CDC, 2018). Besides, the flu, like many other infectious diseases, entails other risks, including the risk of death: it is estimated that between 291,000 and 646,000 people die every year in the world due to flu complications (CDC, 2017). Similar considerations can be made for other vaccines, though with differences as to the probability and the type of medical complications of both the vaccine and the disease in question. For example, there is a very small risk—from 0.087 to 4 (median 2.6) cases per 100,000 vaccine doses—of self-limited and non-life threatening Thrombocytopenic Purpura following measles vaccination (Mantadakis *et al.*, 2010); the measles, mumps, and rubella (MMR) vaccine also entails a small risks of febrile seizures (1 in 1000 doses) and skin rashes with bruise-like spots (1 in 24,000 doses), both of which are anyway more common in cases of measles. But measles can be lethal—1 in 5,000 infected individuals die in high income countries, but the death rate is as high as 1 in 100 in low income countries—and, even when it is not, it can have severe complications, including encephalitis and subsequent permanent brain damage in 1 every 1000–2000 cases in a country like the UK (Oxford Vaccine Group, 2015). Thus, it is safe to say that, from the point of view of risk assessment, vaccination is a rational choice in spite of the small risks it entails, because the individual benefits seem to outweigh some small individual risks. Admittedly, this might not apply to all vaccines. The risk assessment for different infectious diseases is different. When we talk of vaccination here, we only refer to vaccines against infectious diseases whose risks for people’s health and even life are relatively high. While we want to remain neutral as to which vaccines exactly should be included in our discussion, the two examples we have just provided—the flu and measles—have significant risks that make them an appropriate target of our discussion.

If vaccination is based on rational risk assessment, vaccine refusal is in most cases based on irrational risk assessment, although we grant that there might be some broader sense of rationality in which it would still count as rational. In fact, opposition to vaccines is often biased. For instance, often people feel that it is worse, in terms of attribution of responsibility, to be injured by vaccines than by vaccine-preventable infectious diseases, even when the injury is the same and the risk entailed by the disease is higher (Ritov and Baron, 1990). This is an instance of so-called ‘omission bias’, that is, ‘the tendency to see a negative outcome resulting from inaction (omission) as more favourable than the same negative outcome resulting from action (commission)’ (Di Bonaventura and Chapman, 2008: 2). Omission bias is well documented in the psychological literature on vaccination decisions (Ritov and Baron, 1990; Asch *et al.*, 1994; Di Bonaventura and Chapman, 2008). However, some have argued that vaccine denialists are not as irrational as they are sometimes taken to be; instead, they are often part what Mark Navin calls ‘resistant epistemic communities’ that refuse to trust science with regard to the risk assessment of vaccination (Navin, 2015: 31). According to Navin, they are ‘insufficiently committed to truth-oriented inquiry’ (Navin, 2015: 22), an attitude that is not necessarily irrational and that can have different explanations. For example, some feminist approaches would attribute this attitude to what is perceived as a gendered and hierarchical relationship between women who typically care for children and the perceived masculinity of science and doctors who, allegedly, discriminate against women by not involving them in ‘trustful conversation’ (Navin, 2015).

We think this type of accounts, although insightful and more profound than much of the current dismissal of parents’ concerns about vaccination, concedes too much to vaccine denialists: we think that if decision making is not informed by a truth-oriented inquiry, that is enough to make that decision making irrational at least with regard to risk assessment. Navin’s explanation for vaccine refusal certainly suggests that in some cases parents are not blameworthy for their irrational risk assessment, but not that the risk assessment itself is rational. In fact, it is arguments for coercive vaccination policies that are typically taken to be rational because based on considerations of risks: vaccination should be compulsory, so it is sometimes argued, because the state has an obligation to spare children and vulnerable members of a community (e.g. those who cannot be vaccinated for medical reasons) the avoidable risk of suffering and death from infectious diseases or complications thereof (Bambery *et al.*, 2013; Flanigan, 2014; Pierik, 2018). Vaccines produce significant benefits at a small individual cost. In other words, it is both rational and ethically required to take on oneself and to impose on others the small risks of vaccination, because the benefit for individuals and public health is sufficiently large. People who are concerned about the risks of vaccines should be more concerned about the risks of vaccine preventable infectious disease. If they are not, then their risk assessment is irrational, whether or not they are blameworthy for it (and as Navin suggests, they might not be (entirely) blameworthy).

Still, a very small risk does not rule out, and actually entails, that on some occasions some vaccine injury will occur. When this happens, it is very likely that the victim would have remained perfectly healthy, or would at most have caught an annoying infectious disease with no significant complication, without vaccination. This is a consideration that is often evaded in arguments about coercive vaccination policies. We might still be able to justify coercive vaccination policies on grounds of cost-effectiveness or of rational risk taking, but we cannot simply ignore the risk just because it is rational to take it. What if a child is vaccinated as a consequence of a coercive policy and against her parents’ will, and the child is so unlucky as to experience some serious side-effect of vaccines, such as an allergic reaction or a disease like GBS? What are the ethical implications of any episode of iatrogenic disease?

In the next section, we are going to unpack these questions and examine what precise issues they raise. In the analogy: seat belts, risks, and freedom section, we will introduce an analogy between vaccination and seat belt use in motor vehicles, highlighting the policy implications of the analogy. Finally, in on the ethical relevance of risks of vaccine injury for vaccination policy section we are going to explicitly address some of the issues raised in what is the ethical relevance of the risks of vaccine injury section on the basis of that analogy. As we will argue, the analogy suggests that all the issues we have raised so far do not represent strong enough reasons against coercive vaccination policies. While we will not discuss whether the analogy suggests that vaccination should be compulsory or mandatory, our claim is that vaccination policies may and should be coercive, unless enough people decided to vaccinate autonomously and herd immunity were realized—in which case a principle of least restrictive alternative in public health (Childress *et al.*, 2002: 173; Gostin, 2008: 142) would provide a strong reason against coercion. There are different forms that a coercive vaccination policy can take (Giubilini, 2019). Whether the analogy with seat belt requirements suggests that exactly the same type of coercive policy based on fines and other punitive measures for non-compliance is justified is a question that would require a separate discussion. Here, we confine ourselves to the claim that, at the very least, the analogy suggests that some form of coercive vaccination policy is justified.

## What is the Ethical Relevance of the Risks of Vaccine Injury

We have asked how we should ethically assess the realistic possibility that a child is vaccinated as a consequence of a coercive policy and against her parents’ will and the child is so unlucky as to experience some serious side-effect of vaccines. There are actually different issues contained in this type of question.

First, there is an issue concerning the level of coercion that can legitimately be imposed when imposition entails some risks. There are two main reasons why coercive vaccination policies could be enforced: to protect vulnerable people who cannot be vaccinated through herd immunity, and/or to protect the individuals receiving the vaccination. In other words, vaccination policies can be aimed at protecting or promoting either public goods (such as herd immunity) and/or private goods (such as individual immunity). As for the former, risks of vaccines’ side-effects are sometimes taken to be arguments against enforcing policies that are more coercive than what is necessary to achieve herd immunity: it seems unnecessary, and indeed unethical, to expose individuals to some small risks that they (or those making decisions on their behalf) are not willing to take, and that would not benefit others (since we are assuming herd immunity exists), just for the sake of distributing risks fairly (Dawson, 2007). Here, the problem is how much weight we want to give to fairness compared to individual autonomy. We are not going to focus much on this issue here, however, because—and we are turning now to the second reason—it might still be argued that regardless of whether there is herd immunity and regardless of fairness considerations, an individual would still benefit from being vaccinated and there are therefore reasons for exposing her to the small risks of vaccination, given the favourable cost-benefit analysis. If there is no herd immunity, the individual benefit of vaccination is obvious. But even if there is herd immunity, vaccination coverage rates may vary over time and suddenly or the individual might be exposed to communities without herd immunity. Therefore, vaccination is very likely to be very beneficial for the vaccinated individual, regardless of whether at the time of the vaccination there is herd immunity. Thus, in an important sense, vaccinating one’s children is a rational choice even if at present in the area where they are living there is herd immunity. It seems then that there are paternalistic reasons for implementing coercive vaccination policies even if we disregard fairness considerations. Here, the problem is how much weight we want to give to paternalism compared to individual freedom, and particularly the freedom to decide what kind of risks to take on oneself or on one’s children. In any case, with regard to both these points we have just discussed, while it is true that an individual is not at *immediate* risk to others or herself if there are high levels of herd immunity, the preservation of herd immunity is best achieved if each individual is vaccinated because the vaccinated individual reduces *over time* her risk and risk to others.

A second issue concerns a right to compensation for victims of vaccine injuries. One might ask whether individuals who are victims of vaccine injuries should be compensated, for example, through a dedicated and publicly funded compensation fund, which a community could create as a matter of fairness and solidarity towards those who are injured (Mello, 2008). One reason in favour of compensation is that by being vaccinated, someone takes on herself or on one’s children a small risk not only for her own sake or for her children’s sake, but also to benefit others whom she or her children might otherwise have infected: whether or not she has been coerced into being vaccinated or vaccinating her kids, someone who is harmed, or whose children are harmed, for doing something that benefits others could plausibly be entitled to compensation. Addressing this issue would require addressing complicated issues of compensatory justice for which we do not have space. Suffice it to say that there is quite widespread agreement that victims of vaccine-injuries should be compensated through dedicated funds, as already happens in many countries, as a matter of fairness. For instance, in Scandinavian countries there are broad no-fault compensation schemes for both medical treatment and medicines which also include vaccines, while in the UK and the USA there are national schemes that cover childhood vaccines, adult influenza and vaccines given to the armed forces (Looker and Kelly, 2011).

A third issue which we will address is the related issue of the moral and legal responsibility for any vaccine injury. The issue is related because, *if* victims of vaccine injuries are owed compensation, and *if* someone is morally or legally responsible for the injury, then those who are morally and legally responsible for the injury are the ones who are more likely to be under a moral and legal obligation to provide compensation. But the question of moral responsibility and legal liability arises quite independently of the question about compensation, for two reasons. First, victims of vaccine injuries might be entitled to compensation even when no one is morally or legally responsible for the injury, in which case compensation could be obtained through the aforementioned vaccine-injury compensation programmes. Second, those legally responsible for certain injuries could be punished in addition to any compensation they owe to the victims, and the blameworthiness of those morally responsible would not necessarily be nullified by the compensation. Assuming vaccine producers have discharged their obligations to make vaccines as safe as possible, compatibly with efficacy (Vernick *et al.*, 2007: 1994–1995), the question is whether governments should be held morally responsible and legally liable for injuries caused by coerced vaccination. According to Verweij and Dawson (2004: 3124), ‘it might be reasonable that governments accept responsibility for vaccine induced harm’; they suggest that ‘[a]rguably, this should apply if a government assumes that citizens have a moral obligation to accept vaccination ‘for the common good’, and is especially pressing if vaccination programmes are compulsory’. If and when a vaccine injury happens, the victim or the parents of the victim would likely blame and would likely want to take legal action against those who coerced them into vaccinating, especially if they were initially opposed to vaccination. Are these attributions of blame and of responsibility justified?

In the next section, we will argue that the analogy with seat belt requirements provides an answer to this third issue about responsibility for vaccine injuries as well as a more general justification for a paternalistic coercive vaccination policy. The policy is paternalistic in the sense that it aims at protecting those who receive the vaccination regardless of whether they can and do consent, for the reason that vaccination is beneficial to the one who receives it. Granted, there can be reasons for coercive vaccination policies that are not related to harm to others and that go beyond paternalism, such as fairness in the distribution of the burdens of collective responsibilities (e.g. Giubilini, 2019). However, we think it is still important to establish whether the seat belt analogy can also be used in support of mandatory vaccination. Often, as we mentioned above, those who are in favour of coercive vaccination think that if there are no risks to others we should not impose (however small) risks on children or violate parental autonomy just for the sake of fairness (e.g. Dawson, 2007; Flanigan, 2014; Navin, 2015; Pierik, 2018). A paternalistic argument based on the seat belt analogy would strengthen the case for imposing small risks without having to appeal to fairness. Also, as we shall see, even in the case of seat belt requirements the risk of harm to others is a factor to be taken into account and that contributes to the case for imposing small risks on individuals. Thus, the seat belt analogy is not meant to replace arguments based on preventing harm to others and fairness, but to strengthen the case for imposing some small risks on people by appealing to primarily paternalistic considerations. These would hold even if we did not think that fairness is a strong enough reason for coercion.

## The Analogy: Seat Belts, Risks, and Freedom

Wearing seat belts in motor vehicles is compulsory in most countries. All Western countries have some form of seat belt mandates, with the exception of the state of New Hampshire in the USA; most other countries all over the world also have seat belt mandates, though exceptions exist (for instance, Sri-Lanka does not have a national seat belt law) (for a full list, see WHO, 2009). Seat belts can prevent serious injuries and many deaths in car accidents. According to the US Department of Transportation, seat belts laws in the USA, enforced since the mid-80s, reduce the risk of death by 45 per cent and the risk of serious injury by 50 per cent (US Dept of Transportation, National Highway Traffic Safety Administration (NHTSA), 2009). More recent research suggests that seat belts can reduce both fatal and non-fatal injuries by 60 per cent among front seat occupants and by 44 per cent among rear seat occupants (Høye, 2016). Also, primary enforcement—that is, when unbelted drivers can be stopped and ticketed even in the absence of any other offense—reduce fatalities to a greater extent than secondary enforcement—that is, when unbelted drivers can only be ticketed for failure to wear seat belts if they are stopped because of some other offense (Harper *et al.*, 2014). Seat belts also indirectly contribute to a good level of public health by saving resources that would otherwise have to be spent to treat injuries of car accidents. It has been estimated that if New Hampshire had seat belt requirements, 10–20 people could be saved every year.^1^

Some have questioned the efficacy of seat belt requirements by appealing to compensation effects, whereby when people feel safer as a consequence of the enforcement of a safety measure, they become more risk averse, to the point that the additional risks they take might offset the benefit of the safety measure (Adams, 1982; Vrolix, 2006). However, such claims have been disproved by scientific studies (Singh and Thayer, 1992; Cohen and Einav, 2003; Houston and Richardson, 2007), and are now invoked only by small groups opposed to seat belt laws.^2^

However, it remains true that seat belts involve some risks of causing injuries themselves. In particular, a unique injury profile known as ‘seat belt syndrome’ involves intra-abdominal injuries and is often signalled by skin abrasions of the neck, chest, and abdomen; it can involve perforation of the ileum and other internal organ damage that require surgery (Al-Ozaibi *et al.*, 2016). In certain accidents, seat belts injuries are actually more serious than, or as serious as, the injury that would have resulted from the accident if the seat belt hadn’t been used.

Also, sometimes seat belts can increase the risk of injury or even death in a car accident in other ways, because of the dynamics of car accidents. For example, sometimes it would be better if a person were expelled from the vehicle during an accident, or anyway if she could easily get out of the car without having to unfasten the seat belt (which might be very difficult in certain cases, especially if the person is injured or not fully conscious), rather than remaining trapped by the seat belt. Think, for example, of a car that is on fire or that is running towards a cliff or that is sinking into a river: being trapped in the car—e.g. if an injured occupant of the car could not unfasten their seat belts or those of their children—would in such cases increase the risks of dying or injury. Granted, the risk is very small, but it remains true that on some occasions people would be injured or even die as a consequences of wearing seat belts, where that would not have been the case without them.

In spite of such very small risks, and in consideration of the great benefits of seat belt use, today people are normally supportive of seat belt legislations that compel drivers and passengers to wear seat belts or, in case of very young children, to use specific car equipment that is equivalent to seat belts, such as child seats with their own seat belts. A survey in the USA showed that in primary enforcement states, supports for primary enforcement was 71 per cent, but also in States with secondary enforcement the majority of people supported primary enforcement (NHTSA, 2008). However, the introduction of seat belts laws was accompanied by quite a strong opposition. A survey published in 1985, that is, when seat belt laws started to be introduced in the USA, showed that 40 per cent of people in New England were either opposed or strongly opposed to such legislations; besides, before the introduction of seat belt laws, less than 20 per cent people in the USA consistently wore seat belts (Morelock *et al.*, 1985). Nonetheless, seat belt laws were introduced and proved to be extremely effective in increasing seat belt use (Carpenter and Stehr, 2008, Harper *et al.*, 2014).

In Europe, over the decade between 1990 and 2000, when many seat belt legislations were introduced, there was a 24 per cent to 64 per cent (depending on the country) increase in seat belt use among the student population (Steptoe *et al.*, 2002), that is, those on whom legal requirements would have a longer lasting effect. A recent survey in the USA found that 40 per cent of people who do not wear seat belts when sitting in the rear of a vehicle admit that they would wear them if they lived in a state that requires it (Jermakian and Weast, 2018); another survey in the USA found that 91per cent of the respondents ‘always’ wear seat belts and only 1 per cent ‘never’ wear seat belts (Kidd *et al.*, 2014). As often happens, people simply get used to and comply with new legal requirements even when they are initially opposed to them, and in the long run they see it simply as a social norm.

There are libertarian and anti-paternalistic arguments against seat belts laws, well exemplified by the hypothetical dialogue with which we started: simply put, a state has no business in telling me what I can and cannot do, when the consequences of my actions fall only on me or at least to a significantly larger extent on me than on others. This principle seems to apply to seat belt use (Flanigan, 2017). However, one might plausibly reply here that by not wearing seat belts someone will pose an extra cost on the health care system in case she is injured in a way that could have been prevented by the use of seat belts, and therefore it is not true that the consequences of my choice not to wear seat belts fall on me only; this argument, of course, only applies in those contexts where healthcare is publicly funded. Now, Jessica Flanigan suggests that this is not a good argument for mandating certain behaviours: since, on the same egalitarian premises that ground a right to a minimum of healthcare, the right to a decent minimum of healthcare is not waived by one’s bad choices, the freedom to make bad choices is not in conflict with a right to receive healthcare even when healthcare is rendered necessary by bad choices. Thus, since the two rights are compatible, we cannot use the right to use public resources for one’s healthcare as an argument against the right not wear seat belts, even if we agree that not wearing seat belts is a bad choice (Flanigan, 2017).

Engaging with these anti-paternalistic arguments is not the primary goal of this article. The use of public health resources is indeed a relevant consideration when it comes to formulating public health policies, but determining exactly when they are relevant enough and what limitations of individual freedoms they justify requires a separate discussion for which we do not have space. Here we are arguing that these libertarian arguments against seat belt legal requirements—even assuming for the sake of argument that they are valid—do not make a strong enough case against seat belt mandates for children, who cannot make autonomous decisions about whether to wear seat belts and to run certain risks. Since a choice needs to be made on their behalf, the criterion for such choice should be the children’s best interest; if parents do not act in their children’s best interest, they might in some cases be legitimately compelled to do so, for example through mandatory seat belt requirements. Given the efficacy of seat belts at reducing the number of deaths and injuries, compulsory seat belt use for children is justified and indeed an ethical requirement on grounds of best interest. State paternalism may be wrong in the case of adults, but not in the case of children, who actually, by the very same definition of ‘paternalism’, are the proper beneficiaries of paternalistic choices. When parents cannot act paternalistically, it is legitimate for the state to step in.

These considerations about seat belts and seat belt legislations suggest that getting vaccinated or vaccinating one’s children is in many respects, which are relevant for the ethics of policy making, analogous to wearing seat belts or to having one’s children wearing seat belts. While other paternalistic health policies—e.g. smoking bans—could provide effective analogies with paternalistic vaccination policies, seat belt mandates seem to be the most suitable comparison not only because of the historical similarities in terms of the initial resistance to the mandate and because they target primarily non-competent children, but also because we can think of seat belts as a metaphor for vaccination: a vaccine protecting individuals against an infectious disease is like a seat belt protecting individuals in car accidents, or, to push the metaphor a bit, like a seat belt against infectious diseases.

Those who drive regularly are very likely at one point or another to be involved in a car accident; in the same way, people are very likely at one point or another to be exposed to some infectious disease. Wearing a seat belt significantly reduces the risk that the car accident results in serious injury or death; in the same way, in the case of infectious diseases, vaccines significantly reduce the risk that exposure results in serious injury or even death. The fact that people opposed to seat belt use often disregard the facts that are relevant to risk assessment on the basis of mere speculation or poor evidence—such as that seat belts encourage risk taking and therefore increase the number of accident—suggests that they are ‘insufficiently committed to truth-oriented inquiry’ in the same way as many vaccine denialists are, which we have taken to be a sign of irrationality (whether or not they are blameworthy for their irrationality). Also, the fact that when seat belt laws were introduced many people opposed them by appealing to individual liberty or mistaken risk assessment did not stop states from introducing such laws and did not render these laws ineffective; on the contrary, as we have seen, coercive laws have proven very effective both in terms of compliance and of success in reducing injuries and deaths. Thus, adopting a more forward looking perspective, we can say that the fact that coerced vaccination is opposed by those who appeal to individual liberty and to mistaken risk assessment should not stop states from introducing coercive vaccination policies—unless we also think it was wrong to introduce seat belt requirements in spite of public opposition. Granted, the fact that coercion was eventually accepted and proved effective in the case of seat belts is not evidence that the same would happen in the case of vaccination. But at the same time, it does suggest that public resistance is not a reason to think that a coercive policy would necessarily backfire or be politically unfeasible. Resistance to vaccination is very different from resistance to seat belt requirements because there are a number of psychological and socio-cultural factors that explain the phenomenon of vaccine hesitancy or outright vaccine opposition and that would make acceptance of coercive policies more difficult (Callender, 2016). Our point is that resistance itself should not be seen a strong reason against coercive vaccination policies in the same way as it was not considered a strong enough reason against seat belt mandates.

And after all, in the same way as people ended up accepting, complying with, and supporting seat belt mandates, there is a chance people might at least accept and comply with vaccination requirements, provided that they are properly implemented. While a minority will persist in objecting to seat belts or vaccination, there are reasons to believe most people will come to accept them.

And after all, in the same way as people ended up accepting, complying with, and supporting seat belt mandates, there is a chance people might at least accept and comply with vaccination requirements, provided that they are properly implemented. Requirements are properly implemented when, at the very least, there are adequate mechanisms of policing and when the penalty for non-compliance is high enough to deter people from non-compliance. Ideally, however, we might want compliance to be promoted through other means as well, e.g. information campaigns aimed at fostering relationship of trust between parents and healthcare providers.

Coercive vaccination laws, exactly like seat belt legal requirements, can work if properly implemented. For example, in 2017 Italy introduced mandatory vaccination for kindergarten children and compulsory vaccination for school-age children: children could not be enrolled in kindergartens if they were not up to date with the vaccination schedule, and parents of non-vaccinated school-age children were subject to a fine up to 500 euros. The first evidence available suggested that this policy was very effective at raising vaccination rates; according to a study, ‘[th]e increase in VC [vaccination coverage] between 2016 and 2017 (ranging from 0.9 per cent for vaccination against tetanus at 24 months to 4.4 per cent for MMR vaccination at 24 months) was most likely a result of the decree-law being brought into force and supported by the related communication campaign, which was amplified by the media’ (D’Ancona *et al.*, 2018). For example, vaccination coverage for the first dose of the MMR vaccine was 87.2 per cent at 24 months at the end of 2016, and it was 92.2 per cent at 36 months 1 year later, after the introduction of the law. Even if that was still not enough for herd immunity against measles (which is 95 per cent), we need to consider that ‘the effect of the law is likely underestimated as its implementation only began in the second part of 2017’ (D’Ancona *et al.* 2018). Unfortunately, the change of government prevented the law from fully realizing its potential: the new populist government elected in early 2018 had a strong no-vax component that put the enforcement of the law on hold for 1 year and gave voice to the no-vax sentiment among the population. But those preliminary results do suggest that a properly implemented coercive law holds promise for being effective at fostering compliance and raising vaccination rates.

It is also interesting to compare one aspect of the public resistance to seat belt laws—which in an attenuated way persists today—with the current resistance to coercive vaccination laws. As is the case with vaccines, although perhaps more surprisingly, there were and there are even today people who oppose seat belt laws because they claim they were the result of lobbying and blackmailing by automakers—the equivalent of the ‘Big Pharma’ conspiracy theory in the case of vaccine laws (Holdorf, 2002). In a sense, these people are right in the case of seat belt legislations, but their account is only half of the whole story. In the USA, the lobbying by automakers was the result of a deal made by the then Trasportation Secretary Elizabeth Dole, who wanted automakers to push states to pass seat belt laws in exchange for not forcing them to install airbags. The interest of automakers coincided with people’s interest in being protected from serious injuries. The overlapping of interests is also what happens in the case of coercive vaccination policies: the interest of ‘Big Pharma’ in selling more vaccines might well coincide with the interests of individuals and of populations to be protected from infectious diseases. But of course the overlapping of interests was not a good enough argument against seat belts laws in the same way as it is not against coercive vaccination law: the fact that everybody benefits from certain policies is hardly a reason against the policy.

As we have seen in the case of seat belts, when decisions need to be made on behalf of those who are not legally competent to make autonomous decisions, such as young children, the decision ought to be made in their best interest. In the same way as parents ought to be compelled to buckle up their children in their cars because, despite the small risk, buckling up is in their children’s best interest, so parents ought to be compelled to vaccinate their children because, despite the small risks of vaccine, vaccination is in their children’s best interest. There is no reason why the same consideration that is applied in the case of seat belts should not be applied to the case of vaccination.

Besides, non-vaccinated adults and children can infect others, whereas non-buckled up adults pose less of a threat to others. But the analogy with the seat belt case could be sustained also with regard to considerations about harm to others. People who are not buckled up might injure others both directly (their body can be a projectile both within and outside the vehicle) and indirectly (through their consumption of health resources). Thus, the seat belt analogy has something in common with other analogies based on considerations of harm to others that have already offered to justify coercive vaccination laws, such as that between not being vaccinating and randomly gun firing in an open space (Flanigan, 2014) and that between not vaccinating a child and letting her go around with a bottle of toxic bleach that could be ingested by others (Bambery *et al.*, 2013). For instance, unbelted rear seat occupants could slam into the front seat and push the driver into the airbag or steering wheel: a study published in the *Lancet* and based on an analysis of more than 100,000 car accidents concluded that occupants of front seats are five times more likely to die in car accidents if people in the back seats do not wear seat belts, even if they do wear seat belts (Ichikawa *et al.*, 2002). These data both provide reasons for mandating seat belt use also on back seats, as indeed is already the case in most countries, and reinforce the analogy between seat belt requirements and vaccination requirements. However, it might be objected^3^ that the risk of harming others as a consequence of non-vaccination is higher than the risk of harming others as a consequence of failure to wear seat belts, and so the case for compulsory vaccination is stronger than the case for mandatory seat belts. This disanalogy would reinforce rather than weaken the case for mandatory vaccination Based on this disanalogy, we should refine our claim and say that the case for coercive vaccination policies is at least as strong as the case for mandatory seat belt use, if not stronger. We should also bear in mind that there is a way in which failure to use seat belts causes harm to others: through the use of limited health care resources in case of accidents and injuries that seat belts would have prevented. Given the ubiquity of car accidents, this cost would be significant. Of course, this would imply that other policies such as bans on cigarette smoking in favour of vaping might be justified. This might be a reasonable implication. Partly, it will turn on the balance of the public interest and the degree of liberty restriction necessary to reduce harm to others. In the case of seat belts and vaccination, the restriction of liberty is limited.

Many people would perceive that there is a moral difference, in terms of violation of a principle of bodily integrity, between a vaccine requiring piercing of the body and having internal physiological effects on the one hand, and a seat belt that is only applied externally over the intact body on the other. But it is far from clear that this difference has any moral significance beyond some people’s perception. For one thing, when transdermal vaccines are developed (Ita, 2016), this difference would lose much of its (perceived) moral significance. For another thing, both seat belts and vaccines can be thought to protect bodily integrity to the extent that they prevent intrusion into the body of foreign objects that will severely disturb normal physiological function. And in any case, a principle of bodily integrity, like any other principle, is not an absolute one, and can legitimately be sacrificed for the sake of public interest or the best interest of children.

Thus, in virtue of the analogy with seat belt requirements, it seems that if compulsory seat belt laws are justified, then coercive vaccination policies are justified as well. The ethical issues raised by seat belt laws—particularly with regard to risks and benefits and perception thereof, as well as appeals to individual and liberty—are almost identical to those raised by coercive vaccination laws. The only difference is one that makes the case for coercive vaccination laws stronger than the case for coercive seat belt laws, namely the greater threat to others posed by failure to vaccinate than by failure to buckle up. If we think the latter are justified, we have to think the former are justified either, and actually even more strongly justified. The analogy also allows us to address some of the issues that we raised about the ethical relevance of possible side effects of vaccines, as we show in the next section.

## On the Ethical Relevance of Risks of Vaccine Injury for Vaccination Policy

In what is the ethical relevance of the risks of vaccine injury section, we said that the analogy with seat belt requirements provides an answer to the issue about responsibility for vaccine injuries as well as a more general justification for a paternalistic coercive vaccination policy that poses certain risks on individuals

As for the paternalistic aspect, the question is as to how we should balance legitimate paternalism towards child vaccination against individual freedom, and particularly the freedom to decide what kind of risks to take on one’s children or on oneself. The analogy with seat belt laws suggests that paternalism may and indeed ought to take priority, at least in the case of children for whom decisions need to be made. If we think the small risks of seat belt injuries do not represent a strong enough reason not to coerce parents into buckling up their children, then the small risks of vaccine injury do not represent a strong enough reason not to coerce parents into vaccinating their children either.

As for the question about moral and legal responsibility for vaccine injuries, it is important to discuss whether parents can legitimately blame someone or some institution for any vaccine injury, both because of the implications with regard to claims of compensation and because blameworthiness for possible vaccine injuries arguably represents a reason against coercive vaccination policies. The analogy with seat belt laws allows us to see how the idea that governments should be held responsible for any harm caused by the implementation of coercive policies can be challenged. A lot depends on the reasons why the state is forcing individuals to vaccinate or to wear seat belts: if there are institutional responsibilities that require a state to enforce coercive vaccination policies, then the government might have no reasonable choice but to coerce people into vaccinating or wearing seat belts, else it would fail to fulfill its basic responsibilities. Arguably, such institutional responsibilities include the duty to protect vulnerable individuals from vaccine preventable infectious diseases, or the duty to protect individuals—or for libertarians, to protect at least children—from easily preventable car injuries. It might be fair to compensate victims of vaccine injuries through no fault compensation schemes, as described at the beginning. However, the fact that certain individuals have a legitimate claim to compensation for injuries does not entail, either logically or ethically, that someone should be held morally or legally responsible for the injury because they coerced them to take certain risks.

The fact that people have incorrect beliefs or make irrational risk assessment, and more in general that people are not supportive of coercive laws that impose certain risks, does not imply that the state ought not to implement coercive laws, even if a large majority of people are not supportive of such laws. Most of us do think that it was a good thing, and that it was justified, to introduce seat belt requirements in many countries despite initial lack of support, and despite the fact that at least part of this lack of support was due to incorrect risk assessment. Again, we see no reason to think that the case of vaccination should be different.

## Conclusion

Getting vaccinated is like wearing seat belts: both are extremely effective ways of gaining protection against serious injuries and death, for oneself and others. And failing to vaccinate is in many respects like failing to wear seat belts: although both vaccination and seat belt use entail some very small risk of injury, and although both have been and are opposed by many people on grounds of liberty claims, the risk assessment is favourable in both cases. Seat belt use has been made compulsory in most countries. Therefore—so we have argued—vaccination should be legally coerced as well, at least if the objections that are raised against coercive vaccination are the same as the ones that are raised against coercive seat belt laws: risks and liberty infringement.

Even assuming that libertarian and anti-paternalistic arguments provide good reasons for confining seat belt mandates to children, these arguments do not always apply to vaccination, in virtue of the contagiousness of most vaccine preventable diseases and of the possibility of low vaccination rates: in such cases, catching an infectious disease is equivalent not simply to being victim of a car accident, but of being victim of a car accident in such a way as to become a lethal threat to others.

## Footnotes

1. See *New Hampshire Senate Rejects Bill on Mandatory Seat Belting,* New York Times 2007, https://www.nytimes.com/2007/06/01/us/01seatbelt.html .

2. See e.g. this organization at http://www.againstseatbeltcompulsion.org/index.htm#evidence, to give just one example.

3. We are grateful to an anonymous reviewer of this journal for this observation.
